# Effects of air temperature, photoperiod, and soil moisture on leaf senescence and dormancy depth in four subtropical tree species

**DOI:** 10.48130/forres-0025-0007

**Published:** 2025-04-09

**Authors:** Fucheng Wang, Yiming Liu, Heikki Hänninen, Jinbin Zheng, Yu Zhao, Wenwen Chang, Jiasheng Wu, Rui Zhang

**Affiliations:** 1 National Key Laboratory of Forest Food Resources Development and Utilization, Zhejiang A&F University, Hangzhou 311300, China; 2 Provincial Key Laboratory for Non-wood Forest and Quality Control and Utilization of Its Products, Zhejiang A&F University, Hangzhou 311300, China

**Keywords:** Autumn phenology, Day length, Dormancy induction, Leaf colouration, Climate change, Whole-tree ecophysiology

## Abstract

Climate warming has substantially delayed the autumn phenology of trees over recent decades. As the primary focus of previous studies on autumn phenology has been on temperate tree species, the environmental regulation of leaf senescence in subtropical trees under distinct climatic conditions remains poorly understood. To address this gap, using climate chambers, we experimentally examined the effects of air temperature, photoperiod, and soil moisture on leaf senescence and dormancy depth in seedlings of four subtropical tree species. Our results showed that low temperature served as the primary environmental cue driving leaf senescence in all four species, whereas photoperiod and soil moisture had no significant effect on senescence under low-temperature conditions. However, under high-temperature conditions, both drought and short photoperiod accelerated leaf senescence. This suggests that during warm autumns in subtropical regions when the typical senescence trigger (low temperature) is absent, drought and photoperiod are alternative cues to ensure senescence occurs before the onset of winter. Furthermore, we found that leaf senescence and dormancy induction were not closely linked processes. Overall, our experimental results reveal the dominant role of air temperature and its interactions with alternative cues (photoperiod and soil moisture) in regulating autumn leaf senescence in subtropical trees, which challenges the common assumption for a majority of temperate tree species that the primary driver of leaf senescence is short photoperiod. These findings provide valuable insights into the ways trees adapt to subtropical environments.

## Introduction

The global surface temperature is projected to rise at least 1.5 °C above its preindustrial level by 2040 (IPCC 2021). Due to its high sensitivity to air temperature, plant phenology serves as a valuable indicator of climatic warming^[1]^. Phenology determines the length of the growing season and regulates vegetation feedback to the climate by influencing carbon, energy, and water exchange between the terrestrial ecosystems and the atmosphere^[2,3]^. While numerous studies have focused on spring phenology^[4−6]^, comparatively less attention has been given to autumn phenological events, such as leaf senescence, coloration, and fall. Leaf senescence marks a crucial physiological transition in deciduous trees, shifting from active growth to nutrient recycling. This process enables the redistribution of essential nutrients and carbon skeletons to seeds and storage organs^[7]^, while also preparing trees for environmental stresses by optimizing energy use and adapting to seasonal changes^[8]^. Given its ecological importance, further research on leaf senescence is warranted.

In deciduous trees, leaf senescence represents the primary visual indicator of the transition from active growth to dormancy. In boreal and temperate trees, photoperiod, and air temperature are considered two key environmental drivers of leaf senescence^[9−11]^. The primary cue, the shortening of photoperiods, accompanied by declining air temperatures, triggers physiological changes such as reduced photosynthetic activity, growth cessation, and leaf senescence, ultimately leading to dormancy in autumn^[12−16]^. Other factors, such as low summer moisture^[17]^, nutrient availability^[18]^, and soil acidity^[19]^, can also influence senescence. Recent studies have further highlighted the roles of tree productivity during spring and summer^[15]^, as well as summer temperature and precipitation^[15,20]^, in modulating leaf senescence.

Previous research has often relied on statistical relationships derived from long-term observational phenological data collected under natural conditions, either through ground-based monitoring or satellite sensors^[10,21−23]^. However, under natural conditions, multiple environmental factors interact to influence phenology, making it difficult to disentangle their respective contributions. Meng et al.^[24]^ addressed this challenge by analyzing phenological data from the northern Alps, where the unique topography provides varying photoperiods under similar temperature regimes. This natural experimental setting enabled them to isolate the effects of photoperiod and temperature. While effective, such studies remain rare, and controlled conditions are widely regarded as the most reliable approach for determining the relative contributions of different environmental factors to phenological events. Controlled experiments enable researchers to manipulate specific variables, such as temperature and photoperiod, allowing for the decoupling of their effects on leaf senescence and dormancy.

Several studies, using controlled experiments, have demonstrated that in some species, temperature is the dominant factor in leaf senescence. For instance, Heide & Prestrud^[25]^ demonstrated that in a few temperate Rosaceae species, such as apple (*Malus pumila*) and pear (*Pyrus communis*), it is low temperature rather than photoperiod that induces senescence. Wang et al.^[26]^ concluded that low temperature could induce leaf senescence in *Quercus mongolica* and *Larix principis-rupprechtii* even under SD conditions. In contrast, the autumn phenology of many other species, such as *Fraxinus americana*^[27]^ and hybrid aspen^[28]^, has consistently been shown to be driven by photoperiod. Furthermore, interactions of photoperiod and air temperature have also been observed to affect leaf senescence and other autumn phenological processes^[26,29]^. For example, short photoperiod (8 h) accelerates senescence in *Quercus mongolica* and in *Larix principis-rupprechtii* more effectively under low temperature (6, 9 °C) than under high temperatures (18,25 °C)^[26]^*.* Similarly, Koski & Selkäinaho^[30]^ proposed a joint effect model for height growth cessation in boreal *Betula pendula*, where the critical photoperiod for growth cessation increases with greater temperature sum accumulation during the growing season. This suggests that the height growth ceases earlier after a warm summer than after a cool one. Although this model has been validated for height growth cessation in several boreal tree species^[31,32]^, its applicability to other autumn phenological processes, such as leaf senescence in these or other species, remains unknown.

In addition to driving growth cessation and visible leaf senescence, environmental conditions in late summer and early autumn also influence bud dormancy induction. Leaf senescence marks the end of the growing season and is important for trees to survive the severe winter by establishing dormancy. The prevailing view is that short days induce bud dormancy in most boreal and temperate trees, while warm temperatures during the short-day period also increase dormancy depth (i.e., the extent of physiological inactivity in buds, which requires specific environmental cues to resume growth)^[33,34]^. Recently, Wang et al.^[35]^ found that warm autumn temperatures also enhance dormancy depth in subtropical species such as *Torreya grandis* and *Carya illinoinensis*, similar to findings in boreal and temperate trees. This phenomenon is referred to as quantitative dormancy induction^[13]^. However, there is a lack of understanding of the potential connections between leaf senescence and dormancy depth induction, particularly regarding the possibility of these processes occurring synchronously.

While most studies on autumn phenology have focused on boreal and temperate tree species, research on subtropical tree species remains limited. Wang et al.^[35]^ demonstrated that low temperature (LT, + 25/+ 15 °C day/night), as opposed to a high temperature (HT, + 35/+ 25 °C day/night), accelerates the degradation of leaf chlorophyll and leaf senescence in subtropical *Carya illinoinensis*. However, the temperature treatments in their study involved significantly higher temperatures than the typical autumn temperatures in subtropical areas (averaging 10−20 °C). Temperatures below 10 °C have generally been classified as LTs in senescence and dormancy induction studies with boreal and temperate trees^[25,26]^. Additionally, warming autumn temperatures are linked to a greater risk of drought^[36]^. It is therefore essential to determine the impact of the temperatures traditionally classified as LTs on leaf senescence in subtropical trees and to examine the roles of photoperiod and drought in this context. We conducted a controlled experiment applying a fully factorial design to assess the impacts of air temperature, photoperiod, and soil moisture on leaf senescence and dormancy depth in seedlings of four subtropical tree species, thus facilitating the untangling of the individual and interactive effects of these environmental factors. In particular, we hypothesized that: 1) a low temperature is the main environmental factor causing leaf senescence, whereas a high temperature delays leaf senescence; 2) photoperiod and soil moisture play additional roles, with short photoperiods and drought accelerating leaf senescence in high-temperature conditions; 3) a greater dormancy depth is induced by high than by low temperatures, while short photoperiods and drought conditions further induce deeper dormancy under high-temperature scenarios. This experimental study will enhance the understanding of leaf senescence induction in subtropical trees and thereby facilitate the modelling of the phenology and growth of subtropical trees under changing climatic conditions.

## Materials and methods

### Experimental site and plant material

The experiment was conducted in 2020−2021 at Zhejiang A&F University campus in Hangzhou, southeastern China (30°14' N, 119°42' E). Hangzhou has a subtropical monsoonal climate, with a mean annual temperature of + 15.6 °C, mean monthly air temperature of + 4.5 °C in January and + 28.9 °C in July, and mean annual precipitation of 1,614 mm^[37]^. The experiment was implemented with four subtropical tree species, including three native (*Cerasus serrulate, Liriodendron chinense*, and *Sassafras tzumu*) and one introduced (*Carya illinoinensis*) species, all of which are the main afforestation species in the local area. Due to limited space in the climate chamber, small seedlings were utilized for the experiments. For *S. tzumu* and *L. chinense*, first-year seedlings (20 cm tall) were used. For *C. serrulata* and *C. illinoinensis*, second-year seedlings (70−100 cm tall) were used, as their first-year seedlings develop slowly and were not sufficiently mature for examining leaf senescence in this study.

The seedlings were obtained from a nearby nursery of Tianmushan National Forest Station (30°24' N, 119°28' E, Hangzhou, China). The seedlings were propagated by seeds collected from semi-natural (*C. illinoinensis*) or natural (the other three species) forests and were cultivated by standard nursery management practices. On 1 September 2020, the seedlings were transferred to the campus, where the first-year seedlings were transplanted into 1-L polyethylene pots (bottom diameter: 8.8 cm; top diameter: 10.8 cm; height: 14 cm) and the second-year seedlings into 3.8-L (one-gallon) polyethylene pots. For both, a substrate containing five peat: two vermiculite, one perlite, and two organic matter by volume was used (Universal potting soil, Hangzhou, China). The seedlings were kept in natural conditions until the beginning of the experiment on 10 September 2020.

### Experimental design

**Figure 1:**
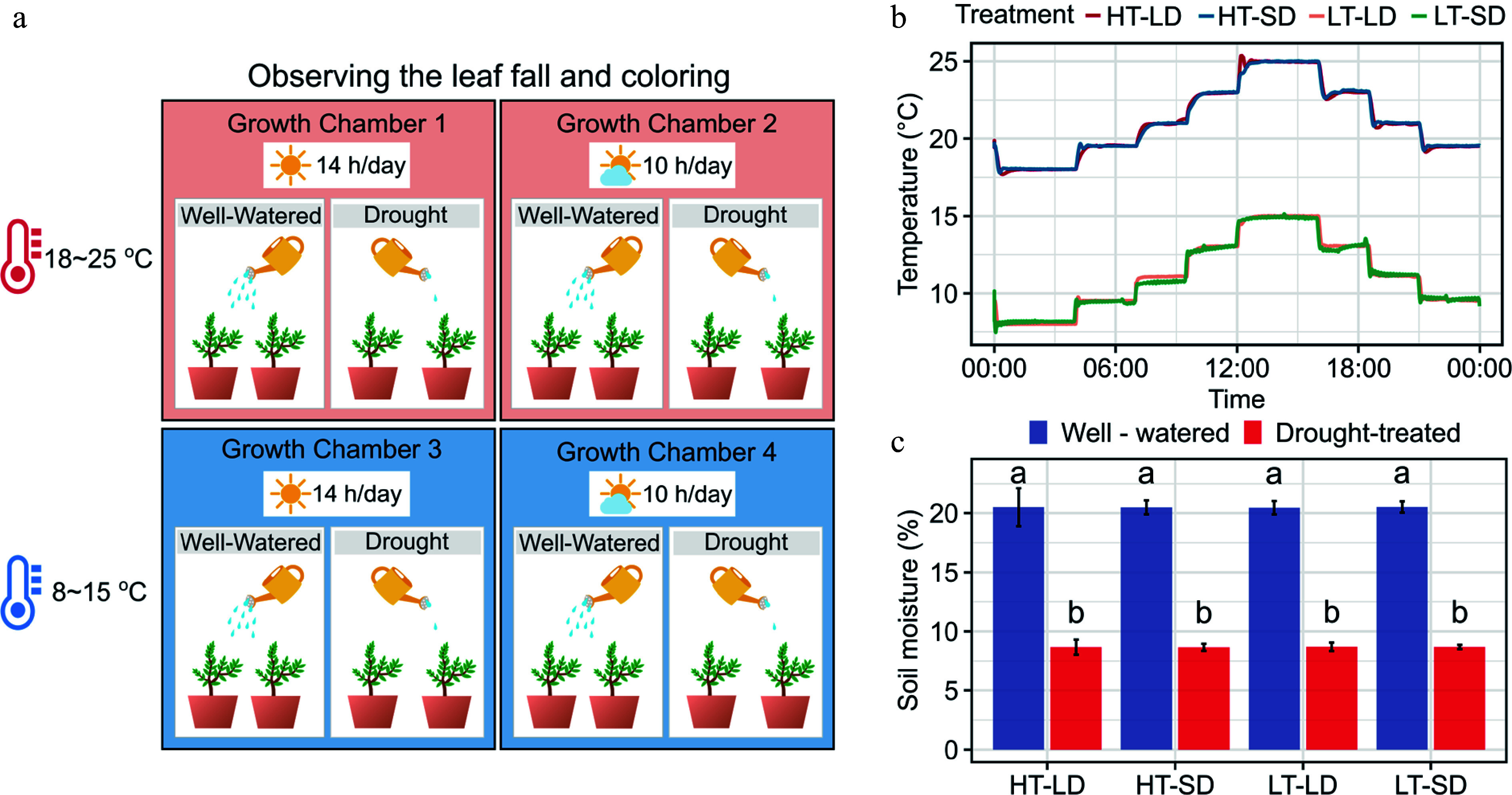
Experimental design of the study addressing the effects of the air temperature, photoperiod, and soil moisture on autumn senescence and dormancy depth in seedlings of four subtropical tree species. (a) The fully factorial 2 × 2 × 2 = 8 design applied in the study, combining two levels of air temperature (HT = 18−25 °C, LT = 8−15 °C), two levels of photoperiod (LD = 14 h day length, SD = 8 h day length), and two levels of soil moisture (well-watered, drought-treated). (b) The daily patterns of the HT and the LT treatment. (c) Soil moisture percentage in the eight treatments at the end of the experiment. The soil moisture percentage was measured for three potted seedlings per treatment. The different letters above the bars indicate a significant difference in the soil moisture percentage between the treatments (post-hoc Tukey's tests, *p* < 0.05).

We established eight treatments in four growth chambers, implementing a 2 × 2 × 2 = 8 fully factorial design based on two temperature, two photoperiods, and two soil moisture treatments (Fig. 1a). The temperature treatments included a high-temperature (HT, fluctuating diurnally from 18 to 25 °C) and a low-temperature (LT, fluctuating diurnally from 8 to 15 °C) treatment (Fig. 1b). The HT and the LT treatments reflected the typical temperature conditions on the study site in September−October and in November, respectively, as derived from a 68-year temperature record from the experimental location Hangzhou, and were applied to approximate warm and cold autumns, respectively. The long-day (LD, 14 h) and the short-day (SD, 10 h) treatments were determined based on the longest and the shortest day lengths occurring on the site at the summer solstice (14.1 h) and winter solstice (10.2 h), respectively. For soil moisture, a well-watered (W) and a drought-treated (D) treatment were applied (Fig. 1c). The details of the soil moisture treatments depended on the air temperature treatment. When combined with HT, the seedlings with W and D treatments were watered every 2^nd^ and every 4^th^ d, respectively, and when combined with LT, W, and D seedlings were watered every 4^th^ and every 8^th^ d, respectively. Furthermore, in each watering, the seedlings in the D treatment received 60% of the irrigation the seedlings in the W treatment did. Soil moisture was measured before each watering. Because of the limited room available in the growth chambers, only six replicates of second-year seedlings (*C. serrulate* and *C. illinoinensis*) were used, whereas, with first-year seedlings (*S. tzumu* and *L. chinense*), the number of replicates was ten. A total of 256 seedlings were used in the experiment. All treatments were implemented in four computer-controlled growth chambers (E-Lotus Technology Co., Beijing, China). In all treatments, relative humidity was 70%, concentration of CO_2_, 400 ppm, and photon flux density PPFD during the light periods was 200 μmol·m^−2^·s^−1^.

The hourly air temperatures inside each growth chamber were monitored with temperature sensors (iButton, Model DS1912L, Embedded Data Systems, Inc., KY, USA; Fig. 1a). Throughout the experiments, the temperatures in the four chambers showed no statistically significant differences between the two HT chambers (21.1 ± 2.33 °C vs 21.1 ± 2.3 °C) or between the two LT chambers (11.3 ± 2.27 °C vs 11.2 ± 2.27 °C). To avoid any systematic differences in the environmental conditions, we rotated the treatments and the respective seedlings between the growth chambers once a week. At the end of the experiment, we randomly selected three potted plants per treatment and compared the W and D treatment seedlings for soil moisture. The results indicated a significant reduction in soil moisture under the D treatment as opposed to the W treatment (Fig. 1c, *p* < 0.05). No significant differences in soil moisture were found between the temperature and the photoperiod treatments.

### Determination of senescence timing

We quantified the leaf senescence process by observing and counting the leaves once a week. If the entire leaf was visually yellow or the leaf had fallen, then the leaf was considered senescent. Since the total number of leaves varied between individuals, we calculated the proportion of leaf senescence for each seedling as the percentage of senesced leaves out of the original number of leaves.

In addition to the senescence observable to the naked eye, we also examined the senescence process by measuring the Chlorophyll Content Index (CCI), a proxy for leaf compositional and spectral attributes such as leaf nitrogen content, chlorophyll a + b concentration, absorbance, reflectance, and greenness. The measurements were carried out with SPAD-502 Plus equipment (Soil Plant Analysis Development, Minolta Camera Co., Ltd, Tokyo, Japan), first every 14 d, and when the leaves started to turn yellow, every 7 d. For each treatment, four leaves in each of the six sampled seedlings were measured. For each measurement, three readings per leaf were taken and averaged to provide a single mean SPAD reading. The SPAD sensor was placed randomly on leaf mesophyll tissue only so that veins were avoided. Because the CCI varied among individuals and species, we calculated the relative CCI for each seedling such that the CCI values were expressed as the percentage of the average measured mean SPAD reading in the measurement out of the maximum mean SPAD reading during the experiment. For the sake of brevity, we refer to the results of the CCI measurements by the concept 'CCI senescence'.

### Dormancy depth measurement

To examine the effects of the experimental treatments on dormancy induction, we assessed the depth of bud dormancy after the treatments were finished on 1 December 2020. We transferred all seedlings from the growth chambers into a warm greenhouse with optimal growing conditions (21.5 ± 2.3 °C and natural photoperiod). We removed the terminal buds from the top of each seedling (approximately, the uppermost 5 cm) and any retained leaves^[38]^. We observed the seedlings twice a week and recorded the date when each of the first three buds showed bud burst. For each seedling, the date of leaf unfolding was recorded as the day when the first leaf was fully unfolded, marked by the visibility of the petiole^[39]^. The dormancy depth was quantified by computing the Growing Degree Hours (GDH) accumulated during the time in the greenhouse required for the leaf unfolding. In autumn, the buds move from shallow para-dormancy to deep endodormancy. A greater dormancy depth (higher GDH values) suggests that a plant is far away from bud burst, whereas a lower dormancy depth (lower GDH values) indicates that the plant is close to bud burst^[6,38]^:

1 $ \mathrm{GDH}=\left\{\begin{array}{l}\Sigma_{t_{\mathit{0}}}^{t_{\mathit{1}}}(T_{hourly}-T_{base}),\quad T\ge T_{base} \\ 0,\quad T\le T_{base}\end{array}\right. $

where, *t*_*0*_ is the date when the seedling was transferred to the greenhouse, *t*_*1*_ is the date when leaf unfolding was observed, *T*_*hourly*_ is the hourly mean temperature, and *T*_*base*_ is a constant set at + 5 °C, representing the minimum temperature threshold required for stimulating budburst^[6]^.

### Statistical analyses

**Figure 2:**
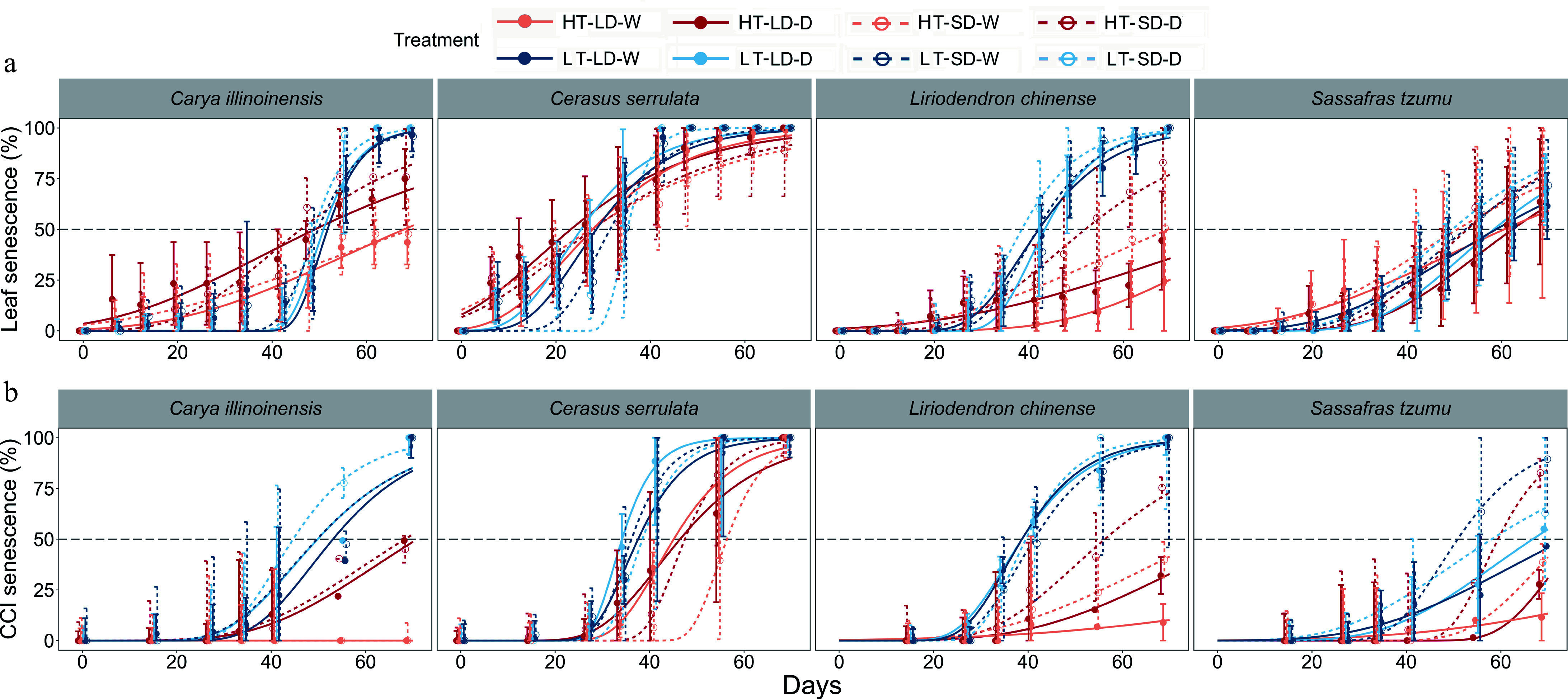
The observed (points) and fitted (sigmoidal curves) time courses of the mean percentages of (a) leaf senescence and (b) CCI senescence (Chlorophyll Content Index, SPAD) in seedlings of four subtropical tree species in a factorial experiment addressing the effects of the air temperature, photoperiod, and soil moisture. For each individual seedling, leaf senescence was determined to occur when 50% of the leaves were either yellow or had fallen, and CCI senescence when the SPAD value decreased by 50% from its maximum value. Treatments: HT = high temperature (18–25 °C), LT = low temperature (8–15 °C), LD = long day (14 h), SD = short day (10 h), W = well-watered seedlings, D = drought-treated seedlings. The error bars represent the standard deviation across individual seedlings in the same treatment.

To examine the progress of leaf senescence (or alternatively, CCI senescence) in the treatment groups, we fitted the Gompertz model to the data representing each treatment group^[26]^:

2 $ {y}={k}_{0}{\exp (- \exp (}{k}_{1}-{k}_{2}{x})) , $

where, *y* is the modelled mean percentage of leaf senescence (or alternatively, CCI senescence) and *x* is the number of days lapsed since the beginning of the experiment. We fitted the equation for each treatment group by using the average of the leaf senescence (or alternatively, CCI senescence) values of the seedlings in each treatment group. The value of parameter *k*_*0*_ was fixed at 1 because the maximum percentage of leaf senescence in the deciduous trees we examined is 100%. Parameter *k*_*1*_ indicates the location of the curve on the horizontal axis without affecting the shape of the curve. Parameter *k*_*2*_ is a coefficient affecting the slope of the curve. The coefficient of determination (*R*^*2*^) of the curve fitted to the leaf senescence and CCI senescence ranged from 0.908 to 0.994 and 0.807 to 0.998, respectively (Fig. 2, Supplementary Table S1). This shows that the Gompertz function enabled us to quantify the average development of both leaf senescence and CCI senescence for all species and treatments included in the study.

To determine the timing of leaf senescence (or alternatively, CCI senescence) in each individual seedling, we also fitted the Gompertz model [Eqn (2)] separately to the data for each individual seedling. Similarly to the first fitting by using the mean value of the seedlings in each treatment group, the value of parameter *k*_0_ was fixed at 1. By using the fitted curve, leaf senescence in each individual seedling was determined to occur when 50% of the leaves were yellow or had fallen, and the CCI senescence when the SPAD value decreased to 50% of its maximal value. To exclude erroneous or low-quality records from the calculation of days to 50 % senescence and to guarantee the representativeness of the results, we omitted the outliers, i.e., measurements deviating by more than 2.5 times the median absolute deviation^[40]^.

For each species, a four-way analysis of variance (ANOVA) was conducted to investigate the effect of the air temperature, photoperiod, soil moisture, and the duration of the treatment until the measurement on the percentage of leaf senescence (or alternatively, CCI senescence). The assessment indicated that the air temperature plays a dominant role in determining the percentage of leaf senescence. Accordingly, when the timing of leaf senescence (or alternatively, CCI senescence) in the individual seedlings was analysed to examine interactions between the temperature and other environmental cues, a three-way ANOVA was carried out. A three-way ANOVA was also used to examine the effects of the temperature, photoperiod, and soil moisture on dormancy depth. A post-hoc Tukey's test was employed to examine pairwise differences at the significance level of *P* = 0.05 in the ANOVA analyses of the timing of leaf senescence (or alternatively, CCI senescence).

We examined the relationship between leaf senescence and dormancy depth by a regression analysis, where the leaf senescence was quantified as the percentage of seedlings showing leaf senescence (50% of the leaves yellow or fallen) after 55 d had lapsed from the start of the experiment. A fixed date well ahead of the end of the experiment had to be chosen for the point of comparison because at or near the end of the experiment the leaf senescence percentage was 100% in the treatments with LT condition.

All statistical analyses were conducted in R version 4.1.3 (R Core Team, 2023)^[41]^.

## Results

**Figure 3:**
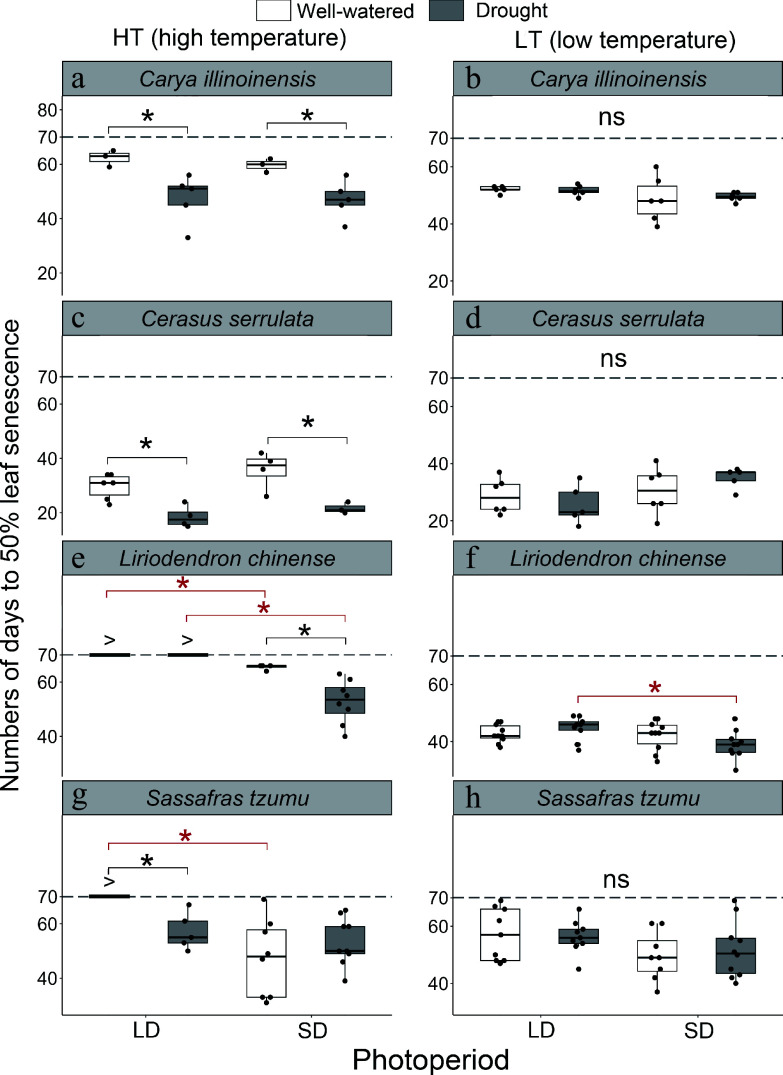
The number of days to leaf senescence in seedlings of four subtropical tree species in a factorial experiment addressing the effects of the air temperature, photoperiod, and soil moisture. For each seedling, the date of leaf senescence was determined as the first day when 50% of the leaves were either yellow or had fallen. The statistics shown were calculated across the seedlings of the treatment group meeting this criterion. If the criterion was met in less than 50% of the seedlings in the treatment group, then leaf senescence was considered not to have taken place in that particular treatment group during the experiment. For those treatments, '>' indicates that the number of days to leaf senescence exceeded the duration of the experiment (70 d). Treatments: HT = high temperature (18–25 °C), LT = low temperature (8–15 °C), LD = long day (14 h), SD = short day (10 h), W = well-watered seedlings, D = drought-treated seedlings. The black and red asterisks indicate a statistically significant difference between the two soil moisture treatments and between the two photoperiod treatments, respectively (*p* < 0.05).

Irrespective of photoperiod and soil moisture conditions, LT treatment advanced both leaf senescence and CCI senescence significantly in all the four tree species examined (Fig. 2, Supplementary Figs S1 & S2; Supplementary Tables S2 & S3). Under LT conditions, the seedlings underwent successive stages of the autumn syndrome, including leaf yellowing and fall (Fig. 2), ultimately leading to 100% leaf senescence and 100% CCI senescence for all species, with a few exceptions, mainly in *S. tzumu*. In contrast, under HT conditions, *L. chinense* and *S. tzumu* leaves either remained green or, when some leaf senescence (or CCI senescence) was observed, it did not even reach 50% in the seedlings before the end of the treatments (Figs 2, 3). Similar analyses were performed for different photoperiod and soil moisture treatments, but no significant effects on leaf or CCI senescence were observed in the SD or D treatments in comparison with the LT treatment. These findings suggest that temperature is the main driving factor regulating leaf senescence in subtropical trees.

**Figure 4:**
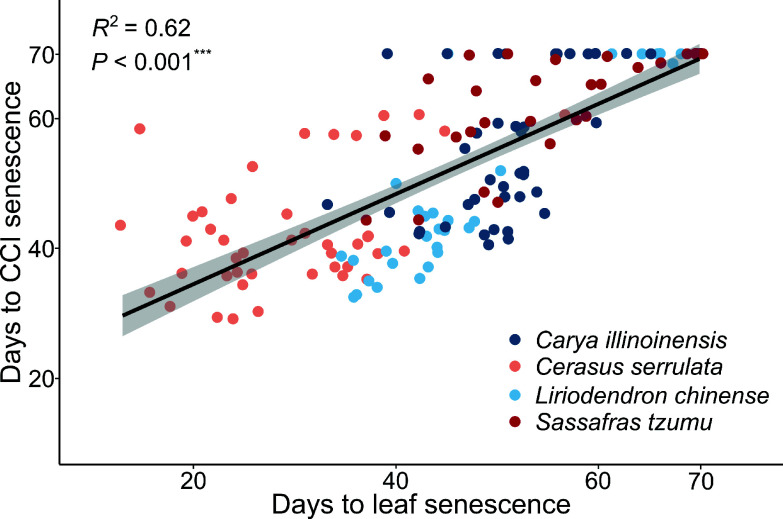
Correlation between the timing of CCI senescence and leaf senescence in seedlings of four subtropical tree species in a factorial experiment addressing the effects of the air temperature, photoperiod, and soil moisture. The leaf senescence date of each seedling was determined as the first day when 50% of the leaves were either yellow or had fallen. The CCI senescence date of each seedling was determined as the day when the Chlorophyll Content Index (SPAD) decreased by 50% from its maximum.

We further explored the interactive effects of photoperiod and soil moisture on the leaf senescence (or CCI senescence) timing under the HT and LT conditions. Given the similarity between the results for leaf senescence and CCI senescence (Figs 3 & 4; Supplementary Fig. S3; Supplementary Tables S2, S4, S5), we focus here on leaf senescence only. Under the HT conditions, several significant interactive effects of photoperiod and soil moisture on leaf senescence timing were observed (Fig. 3, *p* < 0.05). The D treatment advanced leaf senescence in *C. illinoinensis* and *C. serrulata* significantly by an average of 13.8 and 12.2 d, respectively (Fig. 3a & c, *p* < 0.05). In *L. chinense* and *S. tzumu*, more complicated patterns of the interactive effects of drought and photoperiod under the HT conditions were found (Fig. 3e & g; Supplementary Table S2, *p* < 0.05). In *L. chinense*, none of the seedlings showed leaf senescence (50% of the leaves were yellow or fallen) under the LD conditions regardless of the soil moisture condition. However, under SD conditions the D treatment accelerated senescence by 12.7 d, as compared with the W treatment (Fig. 3e). In *S. tzumu*, only the LD condition combined with the W treatment delayed the leaf senescence (Fig. 3g). Under the LT conditions, some interactive effects of photoperiod and soil moisture were seen (Fig. 3b, d, f, h). However, due to the dominance of the effect of a low air temperature, only one significant interaction was found, i.e., in *L. chinense*, the SD treatment accelerated leaf senescence in the D condition (Fig. 3f).

The results found for CCI senescence (Supplementary Fig. S3) were largely consistent with those of leaf senescence (Figs 3 & 4). Due to the dominance of the effect of LT, no significant interactive effects were found for the other two factors under the LT conditions (Supplementary Fig. S3b, d, f, h). Under the HT conditions, SD and D treatment in several cases accelerated the CCI senescence significantly, as compared with LD and W treatment, respectively (Fig. 3a, c, e, g).

**Figure 5:**
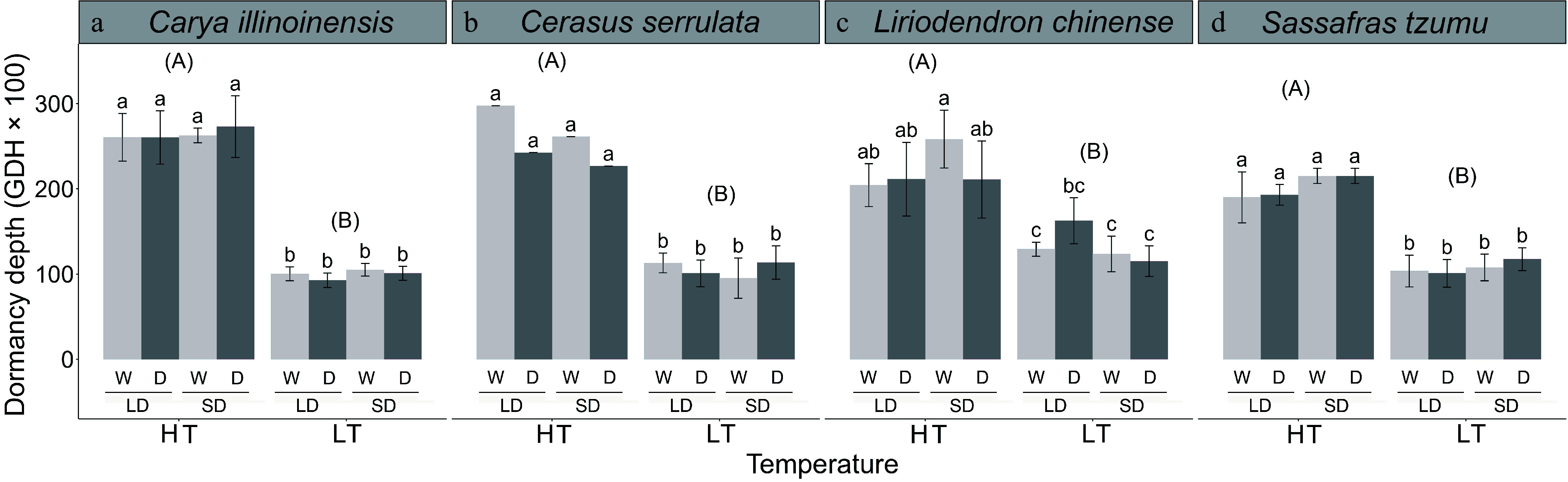
The dormancy depth in seedlings of four subtropical tree species in a factorial experiment addressing the effects of the air temperature, photoperiod, and soil moisture. The dormancy depth was determined after the 70-d treatments as the growing degree hours (GDHs, mean ± standard deviation) required for leaf unfolding under growth-promoting test conditions. The treatments: HT = high temperature (18–25 °C), LT = low temperature (8–15 °C), LD = long day (14 h), SD = short day (10 h), W = well-watered seedlings, D = drought-treated seedlings. The different lower-case letters above the bars indicate a significant difference between the treatments in the mean dormancy depth (post-hoc Tukey's tests, *p* < 0.05). The different upper-case letters indicate a significant difference between high-temperature (HT) and low-temperature (LT) treatments in the mean dormancy depth for data pooled across the two photoperiod treatments and the two soil moisture treatment.

The HT treatment increased the dormancy depth significantly in all four species examined (Table 1). After induction for 70 d, the dormancy depth induced by the HT treatment was higher by 8,865–16,429 GDHs on average than the dormancy depth induced by the LT treatment, varying between the four tree species examined (Fig. 5). With the exception of *L. chinense*, only insignificant effects of the photoperiod and the soil moisture treatment on dormancy depth were found (Fig. 5).

**Table 1:** A three-way analysis of variance for the effects of the air temperature (T), photoperiod (P), soil moisture (SM), and their interactions on the dormancy depth. The dormancy depth was determined after the 70-d treatments in terms of the growing degree hours (GDHs, mean ± standard deviation) needed for leaf unfolding under growth-promoting test conditions.

Treatment	*Carya illinoinensis*			*Cerasus serrulata*			*Liriodendron chinense*			*Sassafras tzumu*	
	F	*P*		F	*P*		F	*P*		F	*P*
T	894.88	< 0.001***		251.60	< 0.001***		106.62	< 0.001***		398.89	< 0.001***
P	1.385	0.246		0.475	0.499		0.002	0.965		12.415	0.001**
SM	0.060	0.936		0.418	0.526		0.178	0.675		0.256	0.616
T × P	0.001	0.980		1.529	0.231		9.416	0.004**		1.948	0.171
T × SM	0.897	0.349		5.333	0.0323*		3.590	0.065		0.041	0.841
P × SM	0.344	0.561		4.144	0.056		7.664	0.009		0.241	0.626
T × P × SM	0.086	0.771		0.115	0.738		0.086	0.770		0.631	0.432
The values shown are the F-statistic of the analysis of variance (ANOVA). ***p < 0.001; **p < 0.01; *p < 0.05. The *p*-values in bold are significant at *p* < 0.05.											

**Figure 6:**
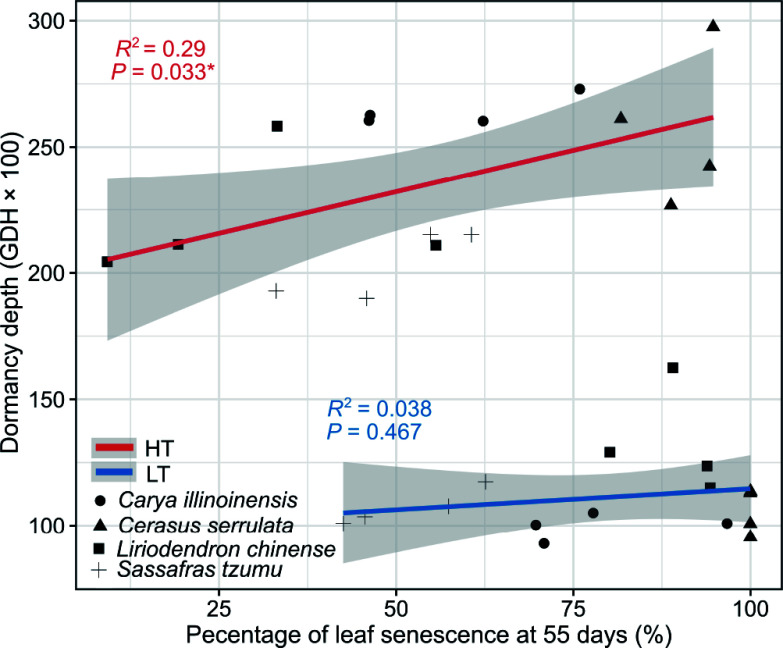
The relation between dormancy depth and the leaf senescence percentage in seedlings of four subtropical tree species in a factorial experiment addressing the effects of the air temperature, photoperiod, and soil moisture. The dormancy depth was determined after the 70-d treatments as the growing degree hours (GDHs, mean ± standard deviation) required for leaf unfolding under growth-promoting test conditions. Leaf senescence was determined to occur in a given seedling when 50% of its leaves were yellow or had fallen. The percentage of seedlings showing leaf senescence in each treatment group was determined after 55 d of the treatment (see Materials and methods). The dependence is shown for data pooled across the two photoperiod and the two soil moisture treatment but separately for treatments with a high temperature (HT, 18–25 °C, red line), and with low-temperature (LT, 8–15 °C, blue line).

The relationship between the dormancy depth measured after 70 d of treatments and the mean percentage of leaf senescence after 55 d from the start of the experiment was different for the two temperature treatments (Fig. 6). The dormancy depth corresponding to any given senescence percentage after the HT treatments was more than twice the dormancy depth after the LT treatments. Furthermore, after the HT treatments, the dormancy depth increased significantly with increasing leaf senescence percentage, whereas no such relationship was observed after the LT treatments (Fig. 6).

## Discussion

### Air temperature as the main driver of leaf senescence and dormancy depth in subtropical trees

In support of our first hypothesis, our results demonstrated that low temperatures play a dominant role in driving either leaf senescence or chlorophyll content index (CCI) senescence across all four subtropical tree species examined (Fig. 2, Supplementary Table S2). Although photoperiod and soil moisture were also examined, only temperature, particularly low temperatures, advanced senescence in all species consistently. This suggests that temperature has a more pronounced impact than the other factors in controlling senescence in these subtropical trees. However, in certain species, such as *C. serrulata*, no significant differences were observed between low-temperature (LT) and high-temperature (HT) treatments in terms of leaf senescence. This may be attributed to the natural abscission between leaves and branches in these species, which facilitates easy shedding^[42]^. Nonetheless, under the same conditions, clear differences were detected in CCI senescence. These findings suggest that in studies of autumnal leaf fall phenology, chlorophyll measurements^[6,14,15]^ offer a more sensitive and reliable approach for detecting subtle differences in the leaf senescence process that may not be easily captured through visual observation, a method commonly employed in large-scale autumn phenology research^[6,15]^. For example, in this study, after about 8 weeks (55 d) of treatment, LT conditions generally advanced CCI senescence by 29.1% to 64.9% compared to HT treatments across the four tested species.

Our results align with previous studies on some temperature-sensitive boreal and temperate species^[6,16,25,26,35]^. For instance, experimental warming by 1.9 °C in October delayed the 50% leaf senescence date of *Fagus sylvatica*, *Quercus robur*, *Betula pendula*, and *Alnus glutinosa* by an average of 11.7 d, as measured using SPAD values^[6]^. Similarly, leaf senescence in *Quercus fabrei* and *Ginkgo biloba* was delayed by 22–30 d on average under autumn warming compared to autumn cooling^[16]^. However, our findings differ from many northern and temperate tree species, where photoperiod is a key driver of leaf fall in these northern species^[26−28,43,44]^. In some species, such as *Populus tremula*^[44]^, senescence is initiated solely by the photoperiod and progresses steadily without any obvious influence of other environmental signals.

Interestingly, compared to the dominant role of photoperiod and the uncertainty in temperature responses of boreal and temperate tree species^[14,25,26,27]^, subtropical deciduous species appear to be more sensitive to temperature. Despite limitations in experimental space, preventing us from testing more species, all four subtropical species exhibited accelerated leaf/CCI senescence under LT conditions. This suggests that low temperature is a critical driver of leaf senescence in subtropical trees. Low temperatures induce a series of metabolic changes that accelerate leaf senescence in plants. These include a decline in photosynthetic efficiency due to the inhibition of Rubisco (a key photosynthetic enzyme) and the accumulation of reactive oxygen species (ROS), which leads to oxidative stress and damages the integrity of chloroplast membranes, thereby accelerating leaf senescence^[45]^.

Additionally, low temperatures trigger a series of hormonal changes, particularly an accumulation of abscisic acid (ABA) and ethylene, which further promote senescence by triggering chlorophyll degradation and leaf abscission^[46]^. Meanwhile, the breakdown of stored carbohydrates for energy, the activation of cell wall-degrading enzymes, and the reallocation of resources to more critical organs further accelerate leaf senescence^[46]^. These findings offer a physiological basis for LT-induced leaf senescence. However, the mechanisms by which LT regulates leaf senescence remain largely unexplored. In subtropical species in particular, the fundamental physiology and molecular mechanisms underlying photoperiod and temperature-regulated leaf senescence still need further in-depth investigation^[47]^.

The contrasting responses of subtropical and boreal tree species may arise from the differences in the seasonality of temperature and photoperiod conditions between these regions^[48]^. In subtropical regions, temperatures around 20 °C commonly occur during autumn. From the perspective of maximizing the length of the growing season, regulating leaf abscission through temperature control offers greater advantages for subtropical tree species^[49]^. This strategy enables subtropical species to prolong their growth during autumns, often warm, thereby optimizing resource utilization before leaf fall. This may explain the occurrence of lammas growth (second flushing, between first and second bud set) in autumn, reflecting a trade-off between the risk of cold damage and carbon sequestration in plants. Lammas growth is common in subtropical species but infrequent in temperate species^[49]^. Due to the shortness and coldness of boreal and temperate autumns^[35,48]^, trees in these more northern zones tend to be more conservative, with photoperiod serving as the more reliable environmental cue for autumn progression.

### Interactive roles of photoperiod and soil moisture in leaf senescence

Our second hypothesis proposed that short photoperiod and drought would play additional roles in driving leaf senescence. This hypothesis was supported for *L. chinense* and *S. tzumu* where photoperiod significantly affected leaf/CCI senescence and for *C. illinoinensis* or *C. serrulata* where soil moisture significantly affected leaf/CCI senescence (Fig. 3, Supplementary Table S2). Overall, our results highlight interspecific differences in autumn leaf senescence among subtropical tree species.

The interspecific role of photoperiod was also found by Wang et al.^[16]^ who reported that SD (10 h) treatments advanced leaf senescence in *Ginkgo* *biloba* significantly by 8 d but had no significant effect on *Quercus fabrei*. Such interspecific variation has been observed in other phenological processes. For example, Zhang et al.^[37]^ found that photoperiod sensitivity of endodormancy release varied among subtropical species, with *L. chinense* showing high sensitivity, whereas other species did not. Besides, Kramer^[12]^ found that *Liriodendron tulipifera*, a related species, is also highly photoperiod-sensitive. The sensitivity of these tree species to photoperiod can be attributed to their evolutionary adaptation to long-term environmental seasonal changes. Both *Liriodendron* and *Ginkgo* are relict trees that have survived hundreds of millions of years and are widely distributed across different climatic zones^[50,51]^. Their long evolutionary history has likely favored the development of mechanisms that allow these species to use regularly changing photoperiod as a reliable environmental cue. This synchronization of physiological processes with seasonal variations ensures that these trees optimize their growth and survival strategies in response to changing environmental conditions. Based on this notion, combined with our research results, we speculate that the tree species of the genus *Liriodendron* and *Ginkgo*, are highly sensitive to photoperiod, possibly at different phenological stages, such as autumnal leaf senescence, winter dormancy release, and spring budburst, which makes them ideal materials for future research on photoperiod sensitivity. Understanding these mechanisms at both the ecophysiological and the molecular level could provide deeper insights into the way these trees adapt to their environments and could offer valuable information for future research on photoperiod sensitivity.

Due to the limited number of climate chambers, we could not set more photoperiod levels to reflect the dynamic photoperiod changes of natural autumn conditions. Instead, the LD and SD treatment were designed to correspond to the longest and shortest photoperiods of the year, which is a commonly used method in photoperiod research^[16,26,29,34]^. This implies that the observed photoperiod effects in this study were likely overestimated relative to natural conditions. However, this does not affect the purpose of this study, which is to qualitatively assess the role of photoperiod. In future studies, if accurate quantification of photoperiod effects on leaf senescence is desired, three or more photoperiod gradients can be set, and a linear fit can be performed using an autumn phenology model^[26]^.

Concerning the role of soil moisture, for all four species, drought interacted with air temperature such that drought (D) treatment accelerated leaf senescence under HT but had no effect under LT (Fig. 3e & g). These results differ from earlier findings in many temperate and boreal species, where drought treatments often had no significant impact on leaf senescence^[52,53]^. For example, Vitasse et al.^[52]^ reported that a drought treatment intercepting 50% of the rainfall did not significantly affect leaf senescence in four temperate species. The greater sensitivity of subtropical trees to drought may reflect their adaptation to warmer autumns, where higher temperatures increase transpirational water loss and amplify drought effects^[54]^. This heightened sensitivity likely reflects an adaptive response to the higher risk of drought during subtropical autumns^[55]^.

Overall, the alternative roles of photoperiod and drought under high but not low temperatures suggest a safety mechanism during unusually warm autumns. While falling temperatures typically drive the transition from active growth to dormancy, short photoperiods, and drought may act as secondary cues to ensure timely dormancy induction during prolonged warm conditions.

### Quantitative dormancy induction and its relationship to leaf senescence

Our third hypothesis addressed quantitative dormancy induction, proposing that deeper dormancy is induced by high temperatures compared to low temperatures. This hypothesis was strongly supported by our findings, which align with previous studies^[33,35,56,57]^. For example, Malyshev^[56]^ reported that a warming of 10 °C for 10 d in October increased dormancy depth in *Fagus silvatica* and *Betula pendula*. Similarly, Wang et al.^[35]^ found that high temperatures induced greater dormancy depth in *Carya illinoinensis* and *Torreya grandis* compared to low temperatures.

Contrary to the second part of our third hypothesis, dormancy depth in three out of the four examined species was influenced solely by air temperature, with photoperiod and soil moisture having no detectable effects. Only *L. chinense* showed sensitivity to both photoperiod and soil moisture. In boreal and temperate trees, dormancy induction is typically controlled by both short photoperiod and temperature^[34,58,59]^. For instance, in hybrid poplar, high temperatures accelerate photoperiod-induced growth cessation and dormancy induction^[34]^. The observed discrepancy between the boreal and the subtropical trees may reflect regional differences in the seasonality of temperature and day length^[48]^.

Leaf senescence and dormancy induction are critical processes for extratropical trees preparing for overwintering. While widely studied independently, their relationship has received limited attention. Our results showed that the dormancy depth after the 70-d treatment was largely independent of the leaf senescence percentage measured after 55 d. Air temperature was the dominant factor regulating dormancy induction, with high temperatures consistently inducing deeper dormancy than low temperatures, regardless of leaf senescence progress (Fig. 6). This suggests that in natural conditions, dormancy depth cannot be reliably inferred from leaf senescence status. Even leafless seedlings may exhibit either deep or shallow dormancy, depending on the prevailing temperature conditions during dormancy induction. This aligns with Malyshev et al.^[60]^, who concluded that leaf senescence is a poor proxy for peak dormancy timing. However, our study measured the dormancy depth only once, at the end of the 70-d treatment period. To better understand the relationship between leaf senescence and dormancy depth, future studies should include repeated measurements of dormancy depth throughout the experimental period^[6]^.

Due to the workload and spatial limitations of experimental work, we examined only four subtropical tree species. Although our results offer valuable insights into the impacts of temperature, photoperiod, and soil moisture on leaf senescence and dormancy induction, the extent to which these conclusions can be generalized to a broader spectrum of subtropical tree species remains uncertain. Further experiments involving a broader range of species are essential for a comprehensive clarification of the relative contributions of the different environmental factors on leaf senescence and dormancy depth and for elucidation of their interrelationship.

## Conclusions

We tested the effects of air temperature, photoperiod, and soil moisture on the leaf senescence and dormancy depth of four subtropical tree species. Our experiments demonstrated that LT is the primary driver of leaf/CCI senescence across all four subtropical tree species examined. SD and drought advanced leaf/CCI senescence under HT conditions, though their influence varied among the species. Under LT conditions, however, SD and drought generally had no significant effects on senescence. Furthermore, our findings provide evidence for quantitative dormancy induction in subtropical trees, indicating that HT conditions contribute to deeper dormancy levels.

In light of the principles of maximizing photosynthesis and optimizing resource utilization before leaf fall, we predict that climate warming may delay autumn leaf senescence in subtropical tree species. However, this effect will be counterbalanced by the increasing drought risk and the effects of decreasing photoperiod. This interplay between environmental factors highlights the plasticity of leaf fall progression in subtropical species. However, further research is needed to better understand the extent and limits of this plasticity, particularly under varying climate scenarios. These results enhance our understanding of the way LT, SD, and drought interact to regulate the leaf senescence of subtropical tree species and also offer valuable insights for modelling future autumn senescence of trees and the carbon sequestration potential of forest ecosystems.

## SUPPLEMENTARY DATA

Supplementary data to this article can be found online.

## Data Availability

The datasets generated during and/or analyzed during the current study are available from the corresponding author on reasonable request.

## References

[1] (2003). Fingerprints of global warming on wild animals and plants. Nature.

[2] (2007). Growing season extension and its impact on terrestrial carbon cycle in the Northern Hemisphere over the past 2 decades. Global Biogeochemical Cycles.

[3] (2014). Net carbon uptake has increased through warming-induced changes in temperate forest phenology. Nature Climate Change.

[4] (2020). Winter temperatures predominate in spring phenological responses to warming. Nature Climate Change.

[5] (2020). Overestimation of the effect of climatic warming on spring phenology due to misrepresentation of chilling. Nature Communications.

[6] (2021). Late to bed, late to rise—warmer autumn temperatures delay spring phenology by delaying dormancy. Global Change Biology.

[7] (2019). Leaf senescence: systems and dynamics aspects. Annual Review of Plant Biology.

[8] (2015). Alteration of the phenology of leaf senescence and fall in winter deciduous species by climate change: effects on nutrient proficiency. Global Change Biology.

[9] (2015). Photoperiod constraints on tree phenology, performance and migration in a warming world. Plant, Cell & Environment.

[10] (2019). A new process-based model for predicting autumn phenology: how is leaf senescence controlled by photoperiod and temperature coupling. Agricultural and Forest Meteorology.

[11] (2022). Warming may extend tree growing seasons and compensate for reduced carbon uptake during dry periods. Journal of Ecology.

[12] (1936). Effect of variation in length of day on growth and dormancy of trees. Plant Physiology.

[13] (1974). Growth and dormancy in Norway Spruce Ecotypes (*Picea abies*) I. interaction of photoperiod and temperature. Physiologia Plantarum.

[14] (2012). Photoperiodic regulation of the seasonal pattern of photosynthetic capacity and the implications for carbon cycling. Proceedings of the National Academy of Sciences of the United States of America.

[15] (2020). Increased growing-season productivity drives earlier autumn leaf senescence in temperate trees. Science.

[16] (2023). Larger responses of trees' leaf senescence to cooling than warming: results from a climate manipulation experiment. Agricultural and Forest Meteorology.

[17] (2023). Earlier leaf senescence dates are constrained by soil moisture. Global Change Biology.

[18] (2022). Delayed autumnal leaf senescence following nutrient fertilization results in altered nitrogen resorption. Tree Physiology.

[19] (2016). The influence of the soil on spring and autumn phenology in European beech. Tree Physiology.

[20] (2023). Increased precipitation leads to earlier green-up and later senescence in Tibetan alpine grassland regardless of warming. Science of The Total Environment.

[21] (2009). Modelling interannual and spatial variability of leaf senescence for three deciduous tree species in France. Agricultural and Forest Meteorology.

[22] (2019). Satellite detection of cumulative and lagged effects of drought on autumn leaf senescence over the Northern Hemisphere. Global Change Biology.

[23] (2020). Modeling leaf senescence of deciduous tree species in Europe. Global Change Biology.

[24] (2021). Photoperiod decelerates the advance of spring phenology of six deciduous tree species under climate warming. Global Change Biology.

[25] (2005). Low temperature, but not photoperiod, controls growth cessation and dormancy induction and release in apple and pear. Tree Physiology.

[26] (2022). Low temperature and short daylength interact to affect the leaf senescence of two temperate tree species. Tree Physiology.

[27] (2019). A spatially explicit modeling analysis of adaptive variation in temperate tree phenology. Agricultural and Forest Meteorology.

[28] (2018). Photoperiodic control of seasonal growth is mediated by ABA acting on cell-cell communication. Science.

[29] (2016). The joint influence of photoperiod and temperature during growth cessation and development of dormancy in white spruce (*Picea glauca*). Tree Physiology.

[b30] 30Koski V, Selkäinaho J. 1982. Experiments on the joint effect of heat sum and photoperiod on seedlings of Betula pendula. Helsinki: Finnish Forest Research Institute. pp. 1−34

[31] (1999). Effects of photoperiod and thermal time on the growth rhythm of *Pinus sylvestris* seedlings. Scandinavian Journal of Forest Research.

[32] (2005). Effects of seed origin and sowing time on timing of height growth cessation of *Betula pendula* seedlings. Tree Physiology.

[33] (2003). High autumn temperature delays spring bud burst in boreal trees, counterbalancing the effect of climatic warming. Tree Physiology.

[34] (2009). Warm temperature accelerates short photoperiod-induced growth cessation and dormancy induction in hybrid poplar (*Populus* × spp.). Trees.

[35] (2022). High autumn temperatures increase the depth of bud dormancy in the subtropical *Torreya grandis* and *Carya illinoinensis* and delay leaf senescence in the deciduous *Carya*. Trees.

[36] (2021). Premature leaf discoloration of European deciduous trees is caused by drought and heat in late spring and cold spells in early fall. Agricultural and Forest Meteorology.

[37] (2021). Chilling accumulation and photoperiod regulate rest break and bud burst in five subtropical tree species. Forest Ecology and Management.

[38] (2020). A simple and convenient method for determination of entrance into dormancy in woody plants. Journal of Berry Research.

[b39] 39Meier U. 2001. Growth Stages of Mono and Dicotyledonous Plants. Berlin: Federal Biological Research Centre for Agriculture and Forestry

[40] (2023). Chilling and forcing proceed in parallel to regulate spring leaf unfolding in temperate trees. Global Ecology and Biogeography.

[b41] 41R Core Team. 2023. R: A Language and environment for statistical computing. Vienna, Austria

[42] (2001). Signals in abscission. New Phytologist.

[43] (1970). Cold resistance and injury in woody plants: knowledge of hardy plant adaptations to freezing stress may help us to reduce winter damage. Science.

[44] (2005). A cellular timetable of autumn senescence. Plant Physiology.

[45] (2018). Effects of chilling on the structure, function and development of chloroplasts. Frontiers in Plant Science.

[46] (2023). Abscisic acid can augment, but is not essential for, autumnal leaf senescence. Journal of Experimental Botany.

[47] (2023). Molecular mechanisms of flowering phenology in trees. Forestry Research.

[48] (2021). A hundred years after: endodormancy and the chilling requirement in subtropical trees. New Phytologist.

[49] (2016). Impacts of bud set and lammas phenology on root: shoot biomass partitioning and carbon gain physiology in poplar. Trees.

[50] (2019). *Liriodendron* genome sheds light on angiosperm phylogeny and species–pair differentiation. Nature Plants.

[51] (2003). The missing link in *Ginkgo* evolution. Nature.

[52] (2021). Impact of microclimatic conditions and resource availability on spring and autumn phenology of temperate tree seedlings. New Phytologist.

[53] (2021). Leafy season length is reduced by a prolonged soil water deficit but not by repeated defoliation in beech trees (*Fagus sylvatica* L.): comparison of response among regional populations grown in a common garden. Agricultural and Forest Meteorology.

[54] (2023). Increased risk of flash droughts with raised concurrent hot and dry extremes under global warming. NPJ Climate and Atmospheric Science.

[55] (2022). Increased drought effects on the phenology of autumn leaf senescence. Nature Climate Change.

[56] (2020). Warming events advance or delay spring phenology by affecting bud dormancy depth in trees. Frontiers in Plant Science.

[57] (2023). Late autumn warming can both delay and advance spring budburst through contrasting effects on bud dormancy depth in *Fagus sylvatica* L. Tree Physiology.

[58] (2010). Temperature-driven plasticity in growth cessation and dormancy development in deciduous woody plants: a working hypothesis suggesting how molecular and cellular function is affected by temperature during dormancy induction. Plant Molecular Biology.

[59] (2018). Storage lipid accumulation is controlled by photoperiodic signal acting via regulators of growth cessation and dormancy in hybrid aspen. New Phytologist.

[60] (2024). The clockwork of spring: bud dormancy timing as a driver of spring leaf-out in temperate deciduous trees. Agricultural and Forest Meteorology.

