# Supplementary material for: Effects of air temperature, photoperiod, and soil moisture on leaf senescence and dormancy depth in four subtropical tree species
Source: For Res (Fayettev). 2025 Apr 9;5:e007. doi: 10.48130/forres-0025-0007 (PMC12141830; doi:10.48130/forres-0025-0007)
Supplement: Supplementary file 1 — Supplementary data to this article can be found online. [file forres-0025-0007-Supplementary.zip › 10.48130_forres-0025-0007-Suppl-FigureS2.pdf]

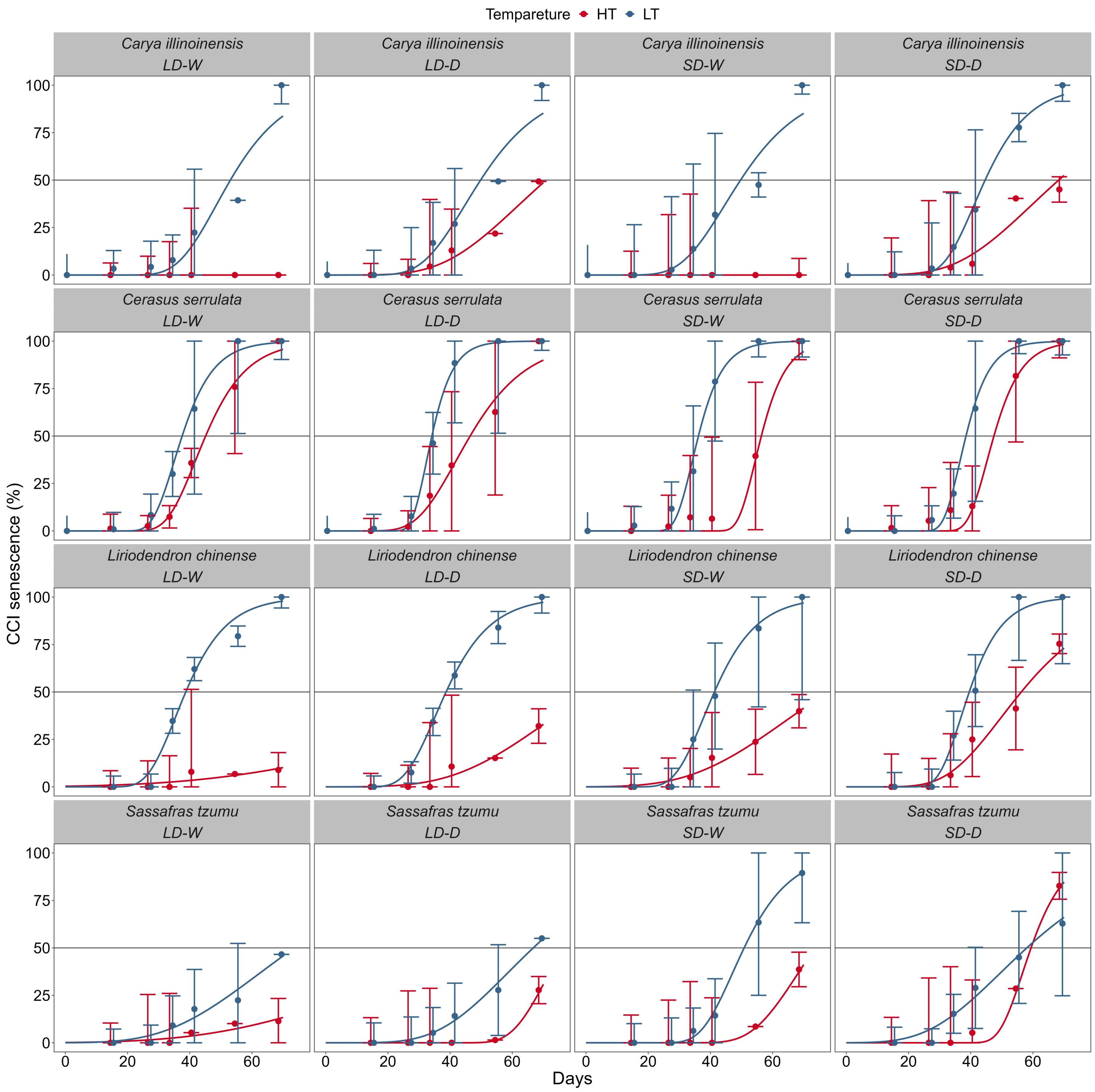

Supplementary Fig. S2. The observed (points) and fitted (sigmoidal curves) time courses of the mean percentages of CCI (Chlorophyll Content Index, SPAD) senescence in seedlings of four subtropical tree species in a factorial experiment addressing the effects of the air temperature, photoperiod, and soil moisture. The results are shown separately for high-temperature (HT: 18 – 25 °C, red symbols) and low-temperature (LT: 8 – 15 °C, blue symbols) treatments as averaged across the seedlings of the indicated combinations of photoperiod and soil moisture treatments represented by the respective panel: LD = long-day (14 h), SD = short-day (10 h), W = well-watered seedlings, D = drought-treated seedlings. The error bars represent the standard deviations across the individual seedlings in the same treatment.
