# Supplementary material for: Effects of air temperature, photoperiod, and soil moisture on leaf senescence and dormancy depth in four subtropical tree species
Source: For Res (Fayettev). 2025 Apr 9;5:e007. doi: 10.48130/forres-0025-0007 (PMC12141830; doi:10.48130/forres-0025-0007)
Supplement: Supplementary file 1 — Supplementary data to this article can be found online. [file forres-0025-0007-Supplementary.zip › 10.48130_forres-0025-0007-Suppl-FigureS3.pdf]

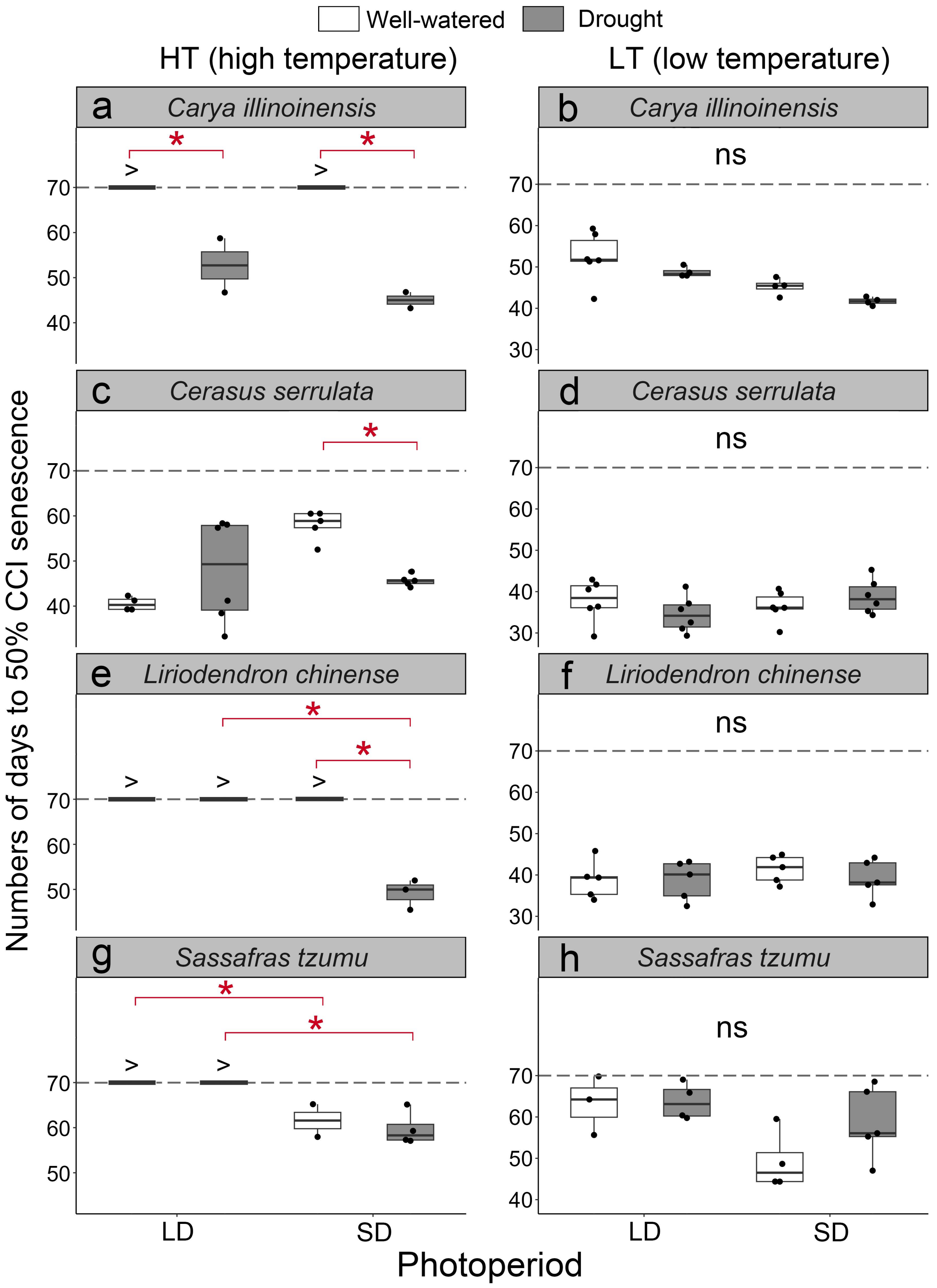

Supplementary Fig. S3. The number of days to CCI (Chlorophyll Content Index, SPAD) senescence in seedlings of four subtropical tree species in a factorial experiment addressing the effects of the air temperature, photoperiod, and soil moisture. For each individual seedling, the CCI senescence date was determined as the date when the SPAD value decreased by 50% from its original value. The statistics shown were calculated across the seedlings of the treatment group meeting this criterion. If the criterion was met in fewer than 50% of the seedlings in the treatment group, then the CCI senescence was considered not to have taken place in that particular treatment group during the experiment. For those treatments the symbol '>' indicates that the number of days to CCI senescence was greater than the 70-day duration of the experiment. The treatments: HT = high-temperature (18 – 25 °C), LT = low-temperature (8 – 15 °C), LD = long-day (14 h), SD = short-day (10 h), W = well-watered seedlings, D = drought-treated seedlings. The red asterisks indicate a statistically significant difference between the photoperiod treatments ($P < 0.05$). For the soil moisture treatments, no statistically significant differences were found.
