# Supplementary material for: Effects of air temperature, photoperiod, and soil moisture on leaf senescence and dormancy depth in four subtropical tree species
Source: For Res (Fayettev). 2025 Apr 9;5:e007. doi: 10.48130/forres-0025-0007 (PMC12141830; doi:10.48130/forres-0025-0007)
Supplement: Supplementary file 1 — Supplementary data to this article can be found online. [file forres-0025-0007-Supplementary.zip › 10.48130_forres-0025-0007-Suppl-TableS1.pdf]

Supplementary Table S1

Parameter values of the Gompertz function fitted to the leaf senescence and CCI senescence data for seedlings of each of the four subtropical tree species examined in the study. The fitting was done separately for each of the eight factorial combinations of the air temperature, photoperiod, and soil moisture treatments by using the average of the leaf senescence (or CCI senescence) values of the seedlings in each treatment group. Treatments: HT = high temperature (18 - 25 °C), LT = low temperature (8 - 15 °C), LD = long photoperiod (14 h), SD = short photoperiod (10 h), W = well-watered seedlings, D = drought-treated seedlings.

Treatment			*Carya illinoinensis*				*Cerasus serrulata*				*Liriodendron chinense*				*Sassafras tzumu*			
			k1	*k2*	R^2	*RMSE*	*k1*	*k2*	R^2	*RMSE*	*k1*	*k2*	R^2	*RMSE*	*k1*	*k2*	R^2	*RMSE*
**Leaf senescence**																		
HT	LD	D	1.214	0.032	0.944	5.491	0.925	0.056	0.969	5.688	1.507	0.021	0.852	4.750	2.625	0.048	0.973	3.632
HT	SD	D	2.078	0.053	0.944	7.143	0.992	0.049	0.964	5.715	2.450	0.054	0.977	4.412	2.788	0.060	0.987	3.305
HT	LD	W	1.594	0.029	0.908	5.311	1.566	0.070	0.989	4.074	2.626	0.033	0.975	1.276	1.439	0.030	0.980	2.786
HT	SD	W	1.255	0.023	0.906	4.972	0.819	0.043	0.965	5.330	1.624	0.029	0.969	2.991	1.668	0.040	0.993	2.115
LT	LD	D	9.423	0.192	0.990	4.780	2.066	0.096	0.965	7.896	5.323	0.131	0.998	2.201	2.871	0.056	0.994	1.940
LT	SD	D	10.000	0.212	0.981	6.631	10.000	0.295	0.981	10.060	4.362	0.123	0.994	3.448	2.496	0.058	0.974	4.844
LT	LD	W	10.000	0.200	0.973	7.858	2.324	0.094	0.940	10.653	3.669	0.096	0.995	2.898	1.899	0.039	0.992	1.943
LT	SD	W	7.712	0.162	0.981	6.955	3.525	0.124	0.962	9.754	4.485	0.116	0.980	6.106	2.489	0.055	0.992	2.455
**CCI senescence**																		
HT	LD	D	2.567	0.041	0.980	2.386	3.345	0.080	0.980	5.220	2.317	0.032	0.959	2.385	6.133	0.085	1.000	0.000
HT	SD	D	2.491	0.042	0.926	5.096	7.415	0.164	0.991	4.667	2.875	0.058	0.978	4.074	7.606	0.134	0.996	2.148
HT	LD	W	NA	NA	NA	NA	4.604	0.110	0.996	2.651	1.744	0.013	0.679	2.278	1.888	0.017	0.860	1.856
HT	SD	W	NA	NA	NA	NA	10.000	0.184	0.991	4.539	2.078	0.032	0.976	2.284	4.635	0.068	1.000	0.071
LT	LD	D	3.256	0.073	0.948	7.988	6.927	0.214	0.998	2.164	3.597	0.102	0.996	2.362	2.612	0.045	0.989	2.024
LT	SD	D	4.215	0.103	0.996	2.518	6.978	0.191	0.998	2.385	5.117	0.139	0.990	4.539	2.135	0.043	0.974	3.795
LT	LD	W	3.911	0.081	0.924	9.563	5.048	0.144	0.997	2.737	3.932	0.111	0.979	5.650	2.081	0.033	0.953	3.484
LT	SD	W	3.207	0.072	0.941	8.412	6.351	0.186	0.992	4.297	4.065	0.107	0.996	2.649	4.651	0.099	0.998	1.672
