# Supplementary material for: Effects of air temperature, photoperiod, and soil moisture on leaf senescence and dormancy depth in four subtropical tree species
Source: For Res (Fayettev). 2025 Apr 9;5:e007. doi: 10.48130/forres-0025-0007 (PMC12141830; doi:10.48130/forres-0025-0007)
Supplement: Supplementary file 1 — Supplementary data to this article can be found online. [file forres-0025-0007-Supplementary.zip › 10.48130_forres-0025-0007-Suppl-TableS2.pdf]

Supplementary Table S2

A four-way analysis of variance of the factors affecting the percentage of leaf senescence and CCI senescence in seedlings of four subtropical tree species in a factorial experiment addressing the effects of the air temperature (T), photoperiod (P), and soil moisture (SM). The time lapsed from the start of the experiment is included as an additional explaining factor in the analysis.

Treatment	*Carya illinoinensis*		*Cerasus serrulata*		*Liriodendron chinense*		*Sassafras tzumu*	
	F	*P*	F	*P*	F	*P*	F	*P*
**Leaf senescence**								
Time	262.530	**<0.001**	747.1	**<0.001**	531.881	**<0.001**	652.900	**<0.001**
T	1.109	0.296	0.157	0.693	99.200	**<0.001**	0.108	0.744
P	0.446	0.506	2.922	0.092	15.074	**<0.001**	10.663	**0.00167****
SM	4.092	**0.0468***	0.262	0.610	4.604	**0.0353***	2.165	0.146
Time *T	160.200	**<0.001**	8.016	**0.006****	98.816	**<0.001**	0.956	0.332
Time *P	0.590	0.445	0.376	0.542	9.443	**0.003****	9.765	**0.0026****
T*P	0.029	0.866	0.068	0.796	4.006	**0.0475***	0.018	0.892
Time *SM	3.970	0.050	0.017	0.897	3.379	0.070	0.253	0.616
T*SM	4.145	**0.0454***	0.131	0.719	2.410	0.125	1.500	0.225
P*SM	0.044	0.835	0.852	0.359	0.373	0.544	0.740	0.392
Time *T*P	0.084	0.772	1.501	0.225	5.507	**0.0217***	0.157	0.693
Time *T*SM	1.432	0.235	0.160	0.691	1.733	0.192	0.066	0.798
Time *P*SM	0.255	0.615	0.394	0.532	0.347	0.558	0.122	0.728
T*P*SM	0.139	0.710	0.0001	0.983	0.146	0.704	0.122	0.728
Time*T*P*SM	0.055	0.816	0.253	0.617	0.37	0.575	0.013	0.909
**CCI senescence**								
Time	112.736	**<0.001**	183.911	**<0.001**	335.066	**<0.001**	144.503	**<0.001**
T	36.437	**<0.001**	9.472	**0.0038***	136.206	**<0.001**	19.065	**<0.001**
P	0.336	0.566	0.512	0.478	3.411	0.074	11.408	**0.00194****
SM	5.432	**0.0249***	0.152	**0.699**	3.644	0.0653	0.658	0.423
Time *T	40.915	**<0.001**	1.585	0.215	74.024	**<0.001**	11.281	**0.00203****
Time *P	0.259	0.6136	0.062	0.804	7.292	**0.011***	16.578	**<0.001**
T*P	0.201	0.656	0.075	0.786	6.979	**0.0127***	0.080	0.779
Time *SM	6.426	**0.0153***	0.057	0.812	3.678	0.0641	0.892	0.352
T*SM	1.621	**0.2103**	0.0102	0.751	10046	0.314	1.560	0.220
P*SM	0.046	0.8311	0.007	0.933	0.404	0.529	0.317	0.577
Time *T*P	0.115	0.7365	0.040	0.842	3.557	0.068	0.137	0.714
Time *T*SM	2.843	0.0995	0.019	0.892	2.485	0.125	4.590	**0.0399***
Time *P*SM	0.055	0.816	0.094	0.761	0.407	0.528	0.025	0.876
T*P*SM	0.007	0.934	0.861	0.359	0.06	0.808	1.859	0.182
Time*T*P*SM	0.004	0.949	0.156	0.695	0.035	0.853	4.630	**0.0391***

The values shown are the F-statistics of the analysis of variance (ANOVA). ***$P < 0.001$; **$P < 0.01$; *$P < 0.05$. *P*-values in bold are significant at $P < 0.05$.
