# Supplementary material for: Effects of air temperature, photoperiod, and soil moisture on leaf senescence and dormancy depth in four subtropical tree species
Source: For Res (Fayettev). 2025 Apr 9;5:e007. doi: 10.48130/forres-0025-0007 (PMC12141830; doi:10.48130/forres-0025-0007)
Supplement: Supplementary file 1 — Supplementary data to this article can be found online. [file forres-0025-0007-Supplementary.zip › 10.48130_forres-0025-0007-Suppl-TableS3.pdf]

Supplementary Table S3

Days to 50% leaf senescence and CCI senescence in seedlings of four subtropical tree species in a factorial experiment addressing the effects of the air temperature (T), photoperiod (P), and soil moisture (SM). Means and standard deviations are based on six or ten replicates of each species and treatment.

Treatment			*Carya illinoinensis*	*Cerasus serrulata*	*Liriodendron chinense*	*Sassafrs tzumu*
T	P	SM				
Leaf senescence						
HT	LD	W	62.3±3.1	29.7±4.6	68.0±1.4	50.0±1.73
HT	LD	D	47.4±9.0	18.5±4.0	67.3±0.6	57.2±6.8
HT	SD	W	59.7±2.5	35.8±6.9	65.5±1.0	47.4±14.2
HT	SD	D	47.0±7.0	21.7±2.1	52.8±8.0	53.4±8.8
LT	LD	W	52±1.2	28.7±6.1	42.8±3.2	57.1±9.9
LT	LD	D	51.7±1.75	25.6±6.8	44.7±4.2	56.3±5.8
LT	SD	W	48.7±7.8	30.5±8.2	42.2±5.2	49.6±8.5
LT	SD	D	49.5±1.5	35±3.67	39±4.9	51.7±10.0
CCI senescence						
HT	LD	W	NA	40.5±1.5	NA	NA
HT	LD	D	52.7±8.5	47.8±11.4	NA	NA
HT	SD	W	NA	58.0±3.3	NA	61.6±5.1
HT	SD	D	45.0±2.5	45.7±1.29	49.1±3.3	59.7±3.8
LT	LD	W	52.4±6.0	37.8±5.1	38.8±4.6	62.2±7.1
LT	LD	D	48.7±1.2	34.5±4.4	38.7±4.8	63.8±4.5
LT	SD	W	45.3±2.0	36.4±3.7	41.4±3.4	49.2±7.1
LT	SD	D	41.7±±1.0	38.8±4.2	39.2±4.5	59.6±8.8
