# Supplementary material for: Effects of air temperature, photoperiod, and soil moisture on leaf senescence and dormancy depth in four subtropical tree species
Source: For Res (Fayettev). 2025 Apr 9;5:e007. doi: 10.48130/forres-0025-0007 (PMC12141830; doi:10.48130/forres-0025-0007)
Supplement: Supplementary file 1 — Supplementary data to this article can be found online. [file forres-0025-0007-Supplementary.zip › 10.48130_forres-0025-0007-Suppl-TableS4.pdf]

Supplementary Table S4

A three-way analysis of variance of the factors affecting the number of seedlings that reached leaf senescence (50 % of leaves yellow or fallen) by the end of the experiment. T = air temperature, P = photoperiod, SM = soil moisture. The analysis was not applied to *Carya illinoinensis* nor to *Cerasus serrulata* because in these species all seedlings reached leaf senescence in all treatments.

Treatment	*Liriodendron chinense*		*Sassafras tzumu*	
	F	*P*	F	*P*
T	45.653	**<0.001**	15.267	**<0.001**
P	8.938	**0.00383****	6.571	**0.0127***
SM	2.637	0.109	0.612	0.437
T*P	9.305	**0.00321****	0.838	**0.0111***
T*SM	2.843	0.0962	0.855	0.358
P*SM	0.092	0.762	0.256	0.614
T*P*SM	0.095	0.759	0.256	0.614

***$P < 0.001$; **$P < 0.01$; *$P < 0.05$. *P*-values in bold are significant at $P < 0.05$.
