# Supplementary material for: Effects of air temperature, photoperiod, and soil moisture on leaf senescence and dormancy depth in four subtropical tree species
Source: For Res (Fayettev). 2025 Apr 9;5:e007. doi: 10.48130/forres-0025-0007 (PMC12141830; doi:10.48130/forres-0025-0007)
Supplement: Supplementary file 1 — Supplementary data to this article can be found online. [file forres-0025-0007-Supplementary.zip › 10.48130_forres-0025-0007-Suppl-TableS5.pdf]

Supplementary Table S5

A three-way analysis of variance of the factors affecting the number of seedlings that reached CCI senescence (50% decrease in the SPAD value) by the end of the experiment. T = air temperature, P = photoperiod, SM = soil moisture. The analysis was not applied to *Cerasus serrulata* because all seedlings of that species reached CCI senescence in all treatments.

Treatment	*Carya illinoinensis*		*Liriodendron chinense*		*Sassafras tzumu*	
	F	*P*	F	*P*	F	*P*
T	109.494	**<0.001*** **	102.400	**<0.001*** **	10.667	**0.0026** **
P	0.041	0.840	1.600	0.215	10.667	0.0026**
SM	4.083	0.504	6.400	0.0165*	2.667	0.1123
T*P	0.012	0.912	1.600	0.215	0.667	**0.420**
T*SM	4.5	0.041*	6.400	**0.0165***	0.667	0.420
P*SM	0.000	1.000	1.600	0.215	0.667	0.420
T*P*SM	0.000	1.000	1.600	0.215	2.667	0.112

***$P < 0.001$; **$P < 0.01$; *$P < 0.05$. P-values in bold are significant at $P < 0.05$.
