# Supplementary material for: Effects of air temperature, photoperiod, and soil moisture on leaf senescence and dormancy depth in four subtropical tree species
Source: For Res (Fayettev). 2025 Apr 9;5:e007. doi: 10.48130/forres-0025-0007 (PMC12141830; doi:10.48130/forres-0025-0007)
Supplement: Supplementary file 1 — Supplementary data to this article can be found online. [file forres-0025-0007-Supplementary.zip › 10.48130_forres-0025-0007-Suppl-FigureS1.pdf]

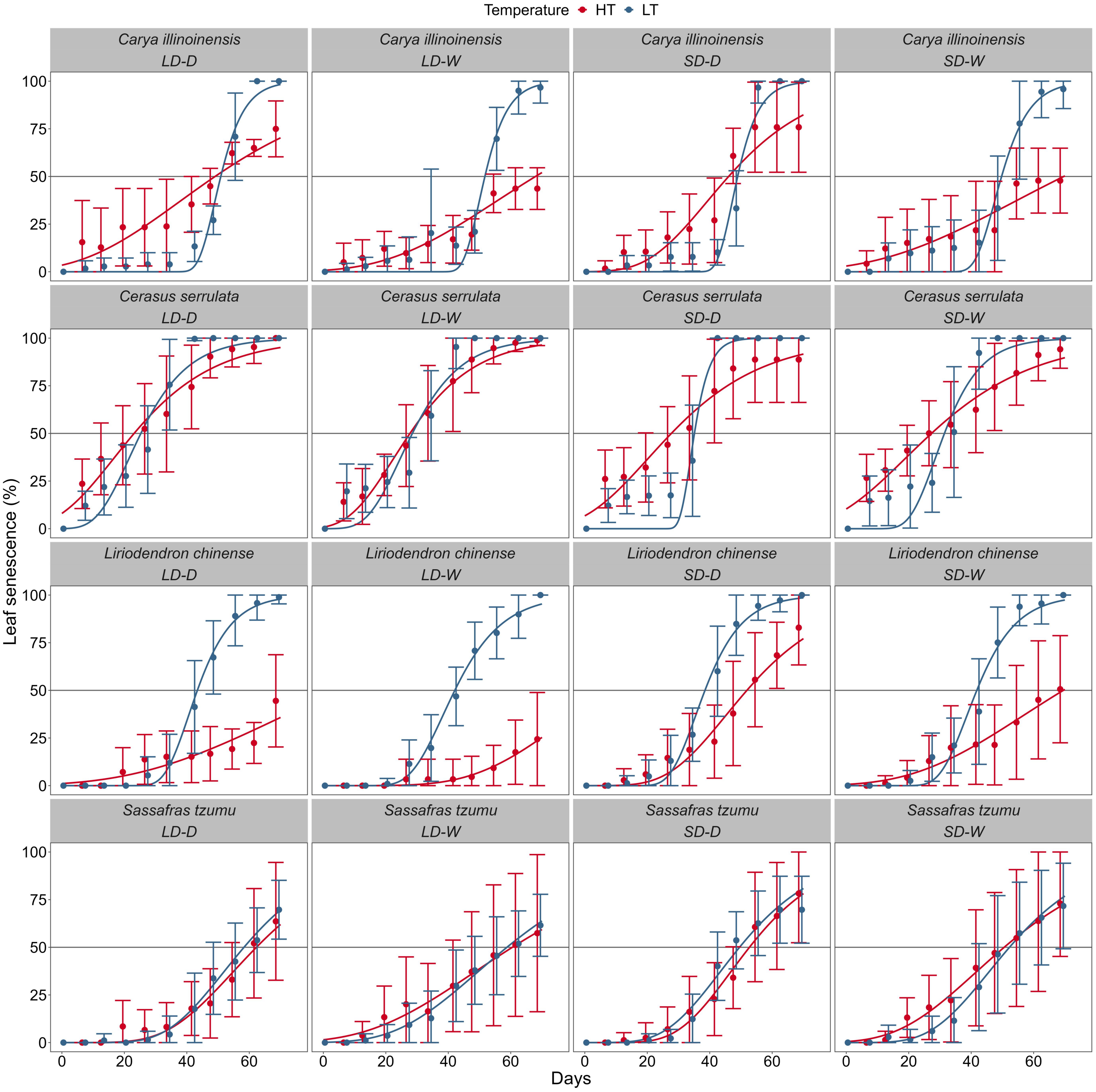

Supplementary Fig. S1. The observed (points) and fitted (sigmoidal curves) time courses of the mean percentages of leaf senescence in seedlings of four subtropical tree species in a factorial experiment addressing the effects of the air temperature, photoperiod, and soil moisture. The results are shown separately for the high-temperature (HT: 18 - 25 °C, red symbols) and the low-temperature (LT: 8 - 15 °C, blue symbols) treatments as averaged across the seedlings in the indicated combinations of photoperiod and soil moisture treatments represented by the respective panel: LD = long-day (14 h), SD = short-day (10 h), W = well-watered seedlings, D = drought-treated seedlings. The error bars represent the standard deviations across the individual seedlings in the same treatment.
